# Acquisition, Visualization and Potential Applications of 3D Data in Anatomic Pathology

**DOI:** 10.15190/d.2016.15

**Published:** 2016-12-31

**Authors:** Shyam Prajapati, Emilio Madrigal, Mark T. Friedman

**Affiliations:** Mount Sinai Health System, Department of Diagnostic Pathology and Laboratory Medicine, New York, NY, USA

**Keywords:** 3D Data, 3D Pathology, Virtual Reality, Anatomic Pathology, Volumetric Data, 3D Scanning

## Abstract

Although human anatomy and histology are naturally three-dimensional (3D), commonly used diagnostic and educational tools are technologically restricted to providing two-dimensional representations (e.g. gross photography and glass slides). This limitation may be overcome by employing techniques to acquire and display 3D data, which refers to the digital information used to describe a 3D object mathematically. There are several established and experimental strategies to capture macroscopic and microscopic 3D data. In addition, recent hardware and software innovations have propelled the visualization of 3D models, including virtual and augmented reality. Accompanying these advances are novel clinical and non-clinical applications of 3D data in pathology. Medical education and research stand to benefit a great deal from utilizing 3D data as it can change our understanding of complex anatomical and histological structures. Although these technologies are yet to be adopted in routine surgical pathology, forensic pathology has embraced 3D scanning and model reconstruction. In this review, we intend to provide a general overview of the technologies and emerging applications involved with 3D data.

## 1. Introduction

Anatomic pathology is an inherently visual field requiring a unique integration of object recognition, medical knowledge, and spatial reasoning to understand and interpret macroscopic and microscopic concepts. These set of skills allow for the capacity to think about three-dimensional (3D) structures and relations from limited information, such as histologic slides, macroscopic photography, and radiographic images. However, drawing conclusions from stationary two-dimensional (2D) abstract representations of one or more complex 3D objects may be particularly challenging.

In the past few years, new technologies have emerged which allow us to acquire, visualize, manipulate, and understand 3D structures. There have been great improvements in computer processors, sensing technologies, graphics rendering, and printing, which have enabled several industries to generate and utilize 3D information. Three-dimensional data refers to the digital representation of objects in the context of three spatial dimensions. Three-dimensional models generated from these data are composed of an object’s geometry and appearance. In the medical field, 3D data more commonly comes in the form of 3D reconstructions of computed tomography (CT) or magnetic resonance imaging (MRI) slices, but may also be seen in 3D models created from surface-scanning of real objects. In anatomic pathology, there has been a progressive utilization of 3D technologies with a slowly growing body of literature. In this review, we first explore the methods of 3D data acquisition and visualization and then discuss the current non-clinical applications and potential clinical applications of this exciting technology.

## 2. Acquisition of 3D Data

An object may be digitally represented as a 3D model, which is composed of the object’s geometry and appearance. The geometry of a 3D model is the mathematical representation of points in three axes (x,y,z) connected to form a 3D structure. Properties, such as color, shading, reflection, images (textures), and animations may be registered to the structure to provide the model with visual details. The final process of combining these characteristics, or even additional models, for visualization, is called rendering.

Whether intended for clinical, educational, or research use, a 3D model may be created by a graphics designer using computer-aided design tools or extracted from real-world image datasets using various radiographic or surface sensing modalities. Essentially all 3D models encountered in the medical field may be classified into two broad categories, surface and solid/volume models, based on the nature of data acquisition and the geometrical characteristics obtained^1^. Surface models use information sensed from the surface of an object to create a shell-like representation of the object. Unlike solid models, surface models are limited to object dimensions and surface characteristics and do not contain internal information. These models are represented by polygonal meshes composed of data points called vertices that are connected to create a series of polygons. Polygonal meshes may be constructed from various surface scanners or even by isolating the boundaries of structures from volumetric data^2^.

**Figure 1:**
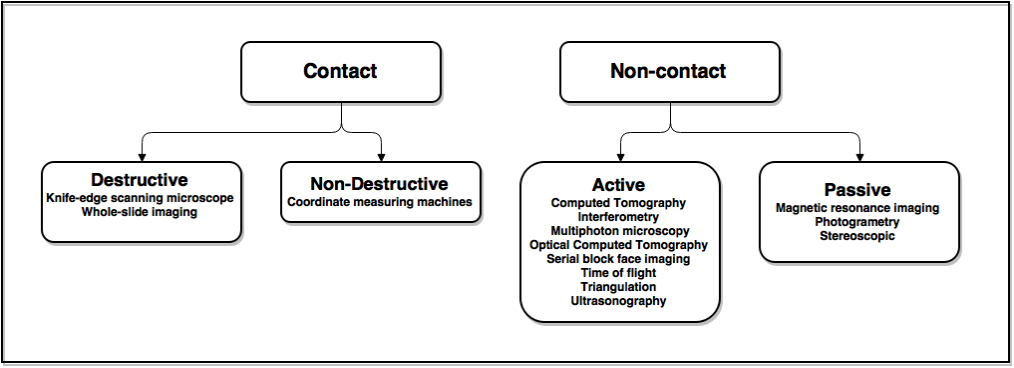
Categorization of scanning techniques used to acquire 3D data.

Rather than creating models from graphics designing software, the medical field benefits most by capturing 3D data from reality, be it surgical anatomic specimens or even the entire human body. The scanning process may involve moving a sensor around a fixed object, using a fixed sensor and rotating an object, or using several sensors that surround an object. The sensing modality can be categorized based on whether the sensor requires contact with the object, emission of radiation, or a specific physical principle^3^ (**Figure 1**). Non-contact techniques rely on actively emitting and then sensing some form of electromagnetic energy, or they are passive and only sense. Contact methods, such as coordinate measuring machines, are used by manufacturers and are not suitable for pliable or delicate objects such as human tissue. Henceforth, we will focus on the more common non-contact scanning methods.

- **3D data in healthcare refers to digital representations of three-dimensional objects and has applications in the surgical, radiology, and pathology communities**
- **There are several methods to obtain 3D data for use in anatomic pathology, which can uncover macroscopic details**
- **Recent advances in graphics processing, display resolutions, and motion sensing have led to the use of virtual reality to visualize and manipulate 3D data in ways like never before**

### 2.1 Active Scanning Methods

Improvements in radiologic imaging and software have played a major role in advancing the methods used to create 3D models from medical data. An important example is CT imaging, which emits high-energy x-rays and measures the radiation transmitted through the object. For over four decades, academic radiologists have successfully developed and implemented algorithms to reconstruct and display 3D volumes from a series of axial CT images^4^. Since then, advancements in CT and MRI methods, in concert with new reconstruction software, have increased 3D reconstruction fidelity and allowed for innovative techniques. It has been shown that modern CT scanners are capable of producing thinner slices and ultimately improve volumetric rendering accuracy^5^. Dynamic CT and MRI techniques have been implemented to capture the route of contrast medium over time to create 3D videos, also known as time-varying data or four-dimensional imaging^4^. Acquiring medical 3D models using radiographic imaging has several advantages over other non-contact scanning techniques. First, radiology equipment is widely available, and it can collect details of internal structures in-situ or from resected surgical specimens^6^. Also, well-established and refined 3D reconstruction algorithms and Digital Imaging and Communications in Medicine (DICOM) standardization enable for quick rendering and interoperability^2^.

Triangulation-based laser sensors cast a laser on a stationary subject and then a camera records the projected laser dot or laser stripe. The positions of the laser emitter and camera are used to calculate (or triangulate) the 3D location of the projected laser as it navigates across the object. The greatest advantage of laser triangulators is their resolution, measured as the smallest distance between two discernable successive points, which can range between 0.05 mm and 0.5 mm^3,7^. They have quick acquisition rates while showing resilience to poor lighting conditions and surface texture effects. Although laser scanners are widely adopted for quality control for manufacturing and reverse engineering, they are costly and use complex equipment.

Structured light-based sensors use a similar triangulation principle as laser triangulation; however, they cast light patterns, such as parallel stripes, rather than lasers. The camera then measures the distortion of the light pattern caused by the object’s varying distance from the light source. This scanning system is highly impacted by environmental lighting and shadows which may lead to inaccurate scans. Structured light-based scanning systems cost much less than triangulation-based scanning systems.

Time-of-flight sensors emit a light pulse, and sensors calculate the surface range by measuring the time it takes for the reflected signal to return. The scanner’s performance is dependent on the distance and size of the object, and therefore scanners are built for various ranges. Long-range time-of-flight scanners perform at the 15 m to 100 m range and can overcome distances limiting other scanning techniques^3^. However, at shorter distances and smaller objects, the sensors become inaccurate due to the difficulty of measuring quick return times in the picoseconds range. Also, time-of-flight sensors also face challenges when dealing with textures which scatter light-reflective surfaces.

### 2.2 Passive Scanning Methods

Photogrammetry and stereoscopic scanning are passive techniques which utilizes digital photographs or video to create 3D models. The process involves a series of photographs or video taken in a 360° manner with overlapping viewpoints. Stereoscopic scanning requires the use of two cameras to triangulate distances. Point clouds are then generated using triangulation principles, which are then used to create a polygonal mesh. Textures are then mapped to the mesh giving the surface of the 3D model a “photorealistic” appearance. Overall, this is an accessible and inexpensive scanning modality, largely due to affordable high-resolution digital cameras, low computing requirements, and open-source or freeware applications (e.g., 123D Catch (Autodesk, San Rafael, CA), PhotoScan (Agisoft, St. Petersburg, Russia), etc.). In recent years, smartphone technologies, including digital sensors and mobile processors have drastically improved. Smartphones may be used as an all-inclusive photogrammetry system where digital images can be captured, reconstructed in 3D (either locally or on remote servers), edited, shared, and viewed on a single device.

### 2.3 Microscopic Scanning Methods

The scanning methods mentioned above may be used to capture macroscopic 3D data. However, there are several emerging technologies which can capture 3D data on the microscopic scale. These methods can change how we visualize human histology and enhance our understanding of microscopic structures. Currently, traditional histologic slides created from formalin-fixed, paraffin-embedded tissue present a 2D cross section, and 3D structure is inferred by the viewer. A volumetric dataset of a series of glass slides can be produced by digitally scanning sequentially deeper levels of tissue using whole-slide imaging technology^8-10^. The 2D digital slide dataset can be then used to reconstruct 3D models using similar computational methods as in CT datasets. Multi-block stitching algorithms which can piece together large portions of tissue that are divided into different paraffin blocks^11^. This multi-block approach, used with automated slide preparation techniques, is a step towards digitally reconstructing entire organs. Although there are different processes to microscopically examine tissue in 3D, this method allows for conventional staining and interpretation.

There are experimental microscopic imaging modalities that forego the tissue processing and staining seen with traditional histologic slides and can be used for 3D reconstructions. X-ray microtomography, also known as micro-CT, can produce volumetric data with resolutions in the submicrometer range; however, it uses high-resolution CT imaging technology^12^. Multiphoton microscopy is an imaging modality that also creates 3D volumetric data at microscopic resolutions, but has the added benefit of being performed in vivo or ex vivo^13^. Clearing procedures can optimize multiphoton images which are nearly indistinguishable from traditional slides. Another volumetric imaging modality, optical coherence tomography, measures reflected near-infrared light and by systematically scanning fresh tissue at depths of 1 to 3 mm^14^.

## 3. Visualization of 3D Data

There are several strategies to digitally visualize the 3D data generated from either the aforementioned acquisition methods or models constructed with 3D computer graphics software. A commonality of digital visualization techniques is that they all essentially require a computing device, 3D rendering software (e.g., Blender, Meshlab, Sketchfab, etc.), display, and for interactivity with the 3D models, peripheral devices are required.

### 3.1 Monitors

**Figure 2:**
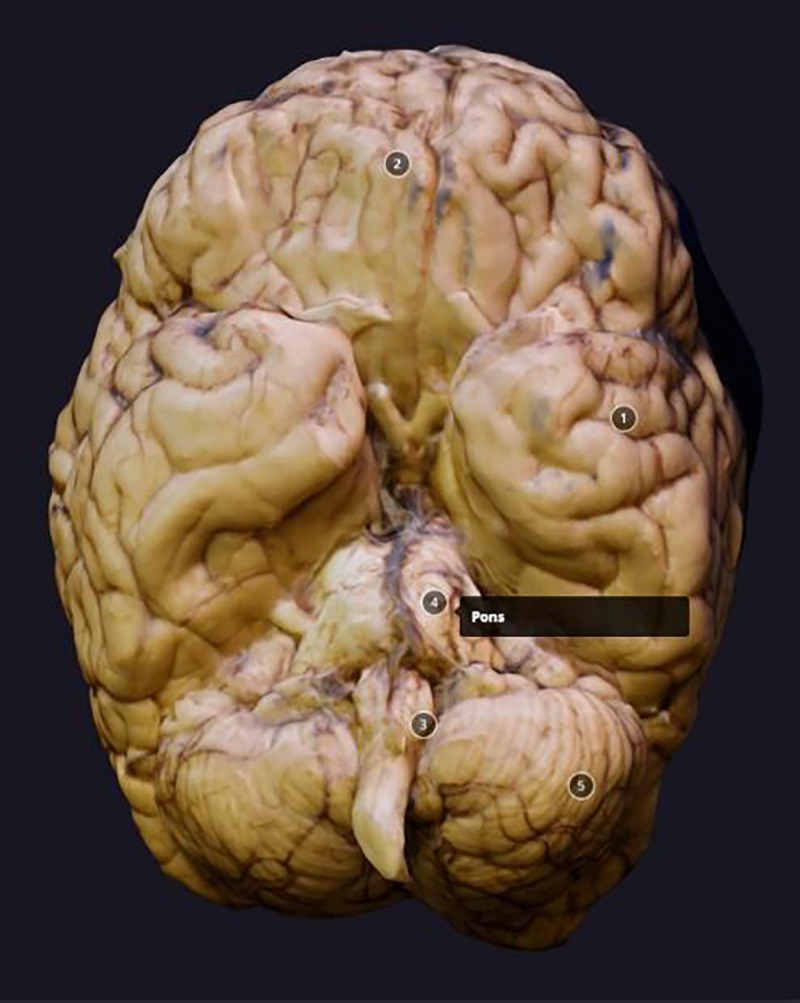
A monoscopic view of a postmortem brain 3D model, scanned using a smartphone (adapted from Normal Brain, In Sketchfab, by S. Prajapati. Retrieved on Nov. 30th, 2016, from https://skfb.ly/TsQy)

The simplest example of a 3D data viewer is a desktop computer and conventional 2D monitor with navigation tools (e.g., image rotation, zoom, and cross section) to appreciate the entire model. Monoscopic 2D displays do not provide an immersive or compelling perception of 3D volume (**Figure 2**). There are commercially available 3D displays which create the illusion of depth^15^. The displays have different techniques in how the content is presented and the equipment required. However, they all use the principle of stereoscopic vision. This works by sending separate images to each eye which converge to give depth perception. 3D monitors require special lenses (e.g., anaglyph, polarized, active shutter lenses), which have their own 3D rendering techniques. Projectors which use these 3D display principals have been used to create semi-immersive environments^16^. Autostereoscopic visualization does not require the use of glasses and works by using a saw-tooth prism on the monitor, which directs left and right images in different directions. Despite these advances, 3D monitors have not been well adopted. The devices themselves and additional equipment have a higher cost than comparable 2D monitors and more importantly some users can experience visual discomfort.

### 3.2 Virtual Reality

**Figure 3:**
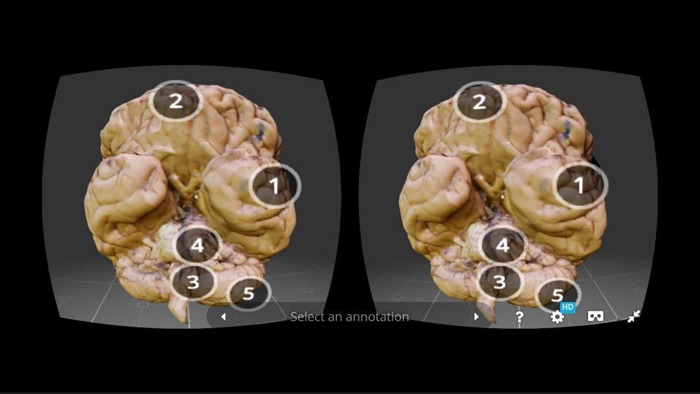
Stereoscopic view of a 3D model as displayed on a smartphone (adapted from Normal Brain,In Sketchfab, by S. Prajapati. Retrieved on Nov. 30th, 2016, from https://skfb.ly/TsQy).

Virtual reality (VR) and augmented reality (AR) are other means to view and interact with 3D data in new and exciting ways. VR typically refers to computer-generated simulations that immerse the user in a 3D environment which provides digital objects a sense of spatial presence. The user experience can be passive, such as in 360° video where the user only has control of the viewing direction. VR can also be active if the user can interact with the artificial environment is responsive to the user's input. To achieve 3D perception, VR devices use a head-mounted display to present side-by-side/stereoscopic images to the user (**Figure 3**).

The past few years have shown several new VR platforms entering the market, including the Oculus Rift (Oculus VR, Menlo Park, CA) and HTC Vive (HTC, New Taipei City, Taiwan). Driving adoption of VR are advances and decrease costs in compact high-resolution displays, graphics processors, and motion sensors (which includes an accelerometer, gyroscope, and magnetometer sensors). Smartphone technology has seen similar technological advances and now mobile VR platforms are emerging, like Daydream (Google, Mountain View, CA) and GearVR (Samsung, Seoul, South Korea). With these mobile VR platforms, the smartphone serves as the computer, display, and motion sensor, and only require low-cost headsets as a chassis. Although VR is a powerful and versatile computing platform for 3D content, it can also induce sickness and visual discomfort. Users are prone to sickness if there is a discordance between what the user sees and the user's vestibular and proprioception systems experience^17^.

### 3.3 Augmented Reality

AR is similar to VR in it uses digital 3D objects which are re-rendered based on the user's perspective, but it does so by supplementing the view of the real world with a layer of 3D content. AR's intended use differs from VR in that it does not immerse the user in an artificial world, but rather enhances the user's real world experience. The blending of real and virtual environments can provide the user with information and visualizations to assist the user with a multitude of tasks. Similar to VR, AR technology has also benefited from recent advances in computing and motion sensing and new devices are becoming available. The HoloLense (Microsoft, Redmond WA) is a head-mounted AR device, which can sense the surrounding environment by using depth cameras and infrared light based structured-light sensors. The mobile platform, Tango (Google, Menlo Park, CA), leverages mobile device cameras and motion sensors to sense reality and then displays a digital overlay using the device's screen. Unlike VR, AR is less prone to induce motion sickness because the user maintains a visual connection with the real world.

### 3.4 3D Printing

Instead of being visualized on screens and digital environments, 3D printers can physically replicate 3D models. 3D printing is an additive process, which begins with a 3D model that is divided into cross sections and converted into a printable file format. The printer then deposits a material (i.e., plastics) to replicate these cross sections, with one layer on top of the other, until the object is formed^7^. Although 3D printers have been available for about the last 30 years, only recently has this technology been adopted by the general public. Printers are now relatively inexpensive with several devices and open-source software to choose from. Today, there is a wide variety of printing materials, including plastic, resin, metals, and ceramics. Also, there are emerging innovations which combine cells, growth factors, and biomaterials to print tissues in a process called bioprinting^18^.

## 4. Applications of 3D Data

The different forms of 3D data content, acquisition, visualization may be utilized in several areas in anatomic pathology. The various applications may be divided into two categories: non-clinical applications, which includes educational and research uses, and clinical applications (**Table 1**). There is a great body of literature discussing the applications of 3D data in disciplines such as medicine, surgery, radiology, and psychiatry, which will not be discussed here^4,18,19^. The following examples do not represent an exhaustive list of 3D data use cases, however, is intended to illustrate the potential these technologies in anatomic pathology.

### 4.1 Educational Applications

In general, medical education has benefited greatly from digital media, including 3D content. Traditionally, text in combination with 2D images and illustrations have been used to teach human anatomy at the undergraduate and postgraduate levels. These methods may fall short in describing complex anatomic structures. Reports have suggested 3D anatomic digital visualizations are associated with improved spatial learning of anatomy.^20,21^ Digital and printed models are the optimal method to convey 3D structure and anatomical relationships, which may be useful for academic presentations and online publications.^22^ They can also be used to teach anatomy, surgical procedures, or macroscopic specimen dissection techniques, without exposing students to the biological and chemical hazards of fresh or formalin fixed tissue.

**Table 1:** Table comparing the clinical vs. non-clinical applications of 3D data in anatomic pathology

Educational Applications	Research Applications	Clinical Applications
Anatomy education	Histology-based research	Documentation of surgical specimens
Histology education	Histologic morphology and "omics" data correlational research	Multidisciplinary conferences
Gross dissection training		Whole-slide imaging evaluation
Academic presentations and publications	Macroscopic and microscopic correlational research	Documentation of forensic evidence

A wide variety of visualization methods have been introduced to anatomy education. These technologies can display anatomic structures and incorporate interactivity, which cannot be achieved through print alone. Digitally rendered 3D anatomical models can rotate, zoom, and remove obscuring layers to make structures visible from any angle and may be accompanied by text descriptions, annotations, and quizzes^20,23^. The Sectra Table (Sectra, Linköping, Sweden) is a large, multi-touch display, which allows users to interact with 3D volumes of real bodies rendered from CT and MRI scans. Using the touch screen allows for a "virtual knife" tool that creates cross sections to reveal internal structures. VR has also been applied to teach anatomy by using what is essentially a virtual anatomy atlas (e.g., 3D Organon VR Anatomy). More recently, a stereoscopic recording technique was used to create an immersive macroscopic dissection tutorial to teach anatomic pathology residents at a single institution^17^. The passive VR experience was well accepted, and residents did not show significant motion sickness. Applications of AR in anatomy education were introduced in the 1990s, which essentially displayed 3D bones over their real counterparts^24^. These early prototypes have come a long way, and modern AR tools offer impressive visualizations of 3D models. HoloAnatomy (Case Western Reserve University, Cleveland, OH) is an application developed for the Microsoft HoloLens, which displays an interactive 3D anatomical model.

3D Printing has also been used for pathology education and has the potential to recreate almost any complex anatomy. Mahmoud et. al. described a method to created realistic 3D models from surgical resection specimens^25^. Models are scanned using photogrammetry at the gross dissection bench, then assembled and edited using 3D graphics software. The edited 3D models are printed using a commercially-available multicolor 3D printer. The models printed were durable and able to display macroscopic lesions found in the original specimen. Inconsistent colors, inability to reproduce fine surface textures, and size restrictions were limitations described by their experience.

### 4.2 Research Applications

Several areas of anatomic pathology research have emerged using 3D data, and it has been used to enhance our understanding of normal and pathologic histology. Tissues are inherently 3D and traditional histologic methods only represent cross sections of microscopic structures, but volumetric histologic data can overcome this as mentioned above. 3D histologic models may be used to better investigate tumor growth patterns in 3D space and display tumor heterogeneity^8^. Capsular invasion of thyroid follicular neoplasms and visualization of lymphatic structures in breast cancer patients have added to our knowledge of these diseases^26,27^. 3D reconstructions may be coregistered with various radiology methods to add histologic details to macroscopic images^28^. Staining techniques and "omics" data can be coregistered to 3D histologic models to bridge the gap between histological morphology and molecular correlation.^10,29^.

### 4.3 Clinical Applications

There are numerous clinical applications of 3D data currently in use by radiology and surgery. On the other hand, anatomic pathology has seen very limited clinical use and rare adoption. This may be due to the limited experience of using these technologies by pathologists, government regulations, lack of reimbursement, and unclear utility. Routine use of microscopic and macroscopic 3D data in diagnostic pathology are far from becoming a reality, but the potential applications are emerging.

It is possible to routinely acquire 3D data from surgical specimens. The Pathobin 3D System (Pathobin, Parkville, Australia) is a photogrammetry-based scanning device developed specifically for photo-realistic 3D scanning of specimens. To scan, the specimen is placed on a turntable that rotates as a camera captures a 360° view of the object. The image capturing occurs in a matter of seconds; however, the 3D model requires several minutes to be constructed. 3D models can potentially replace traditional 2D photography and preserve the entire external view even after the specimen is discarded. Following scanning, the models may be annotated to display anatomical landmarks and the location of sampled sections. Also, measurements can be taken directly from 3D models and can capture some information useful for cancer staging. Scanned cases may be then used to resolve gross dictation errors, create educational collections, present at multidisciplinary conferences, and for medical-legal documentation. Since photogrammetry can only scan surface information, internal and obscured structures may not be acquired.

3D information obtained at the microscopic level can assist in evaluating tissue morphology in the clinical setting, especially when interpreting the complex architectural patterns such as in the diagnosis and prognosis of cancer lesions^30-33^. Bucur et al. looked at 3D reconstructions of breast tissue to evaluate for in situ and invasive carcinoma^30^. They concluded that the visualizations were highly informative and performed well in discriminating normal tissue from carcinoma. Maruyama et al. used whole slide imaging scans to create 3D reconstructions, which were then used to investigate oral squamous cell carcinoma^31^. In this study, the tumor fronts viewed by conventional 2D glass slides were interpreted as having multiple foci of invasion but were then seen as a continuous invasion in 3D reconstructions. Similarly, few studies have aimed to show the prognostic significance of 3D microscopic data. For example, 3D visualizations obtained from pT1 colorectal cancer cases were used to investigate the spatial characteristics of colonic submucosal vessels and its role in lymphovascular metastasis^33^. Their findings suggested that the depth of tumor invasion into the submucosa correlated less with the occurrence of metastasis in comparison to the volume of submucosal invasion. These few examples showcase the emerging applications of microscopic 3D data to human diseases. However there is scarce data supporting the utility and value of these technologies in routine use.

Image analysis algorithms extract meaningful information from digital datasets. It has demonstrated the potential to assist pathologists in automated evaluation of 2D whole-slide images^34,35^. The same principles can be applied to 3D surface and volumetric data. Algorithms have been created for tumor measurement and cellular morphology analysis with diagnostic and prognostic implications^36,37^. Content-based image retrieval, also known as reverse image search or high-dimensional visual information retrieval, is an emerging application of image analysis, where the search query is a multidimensional dataset and not text^38,39^. Shapes, textures, and other visual features of spatial or spatial-temporal microscopic/ macroscopic data are characterized by computer algorithms and search for similar objects in a repository. The search retrieves the most similar object with contextual data to essentially provide a suggested diagnosis. Though none of these image analysis tools have been approved for clinical use, they may reshape how pathologists.

VR headsets may be used to display whole-slide images for diagnostic evaluation, but did not show added benefit in comparison to traditional 2D monitors^40^. A typical real life digital pathology workstation consists of several monitors for displaying slides, managing cases, and reporting results, as well as, a peripheral device to navigate through digital slides. It is possible to simulate this workstation via VR and AR and thus reducing the equipment and space occupancy requirements of a workstation.

Interestingly, forensic pathology has embraced 3D data acquisition and modeling with promising results. Documentation of forensic evidence historically consisted of a collection of written protocols, photographs, sketches, radiographic imaging, and pathology reports. The use of 3D surface scanning and radiographic reconstructions has been reported to collect information on the injuries of the victims and objects involved in the crime^41,42^. Also, the surface of large objects such as cars and even rooms in which a crime occurred, may be digitally scanned. Static forensic data has been taken a step further by using computer animation techniques to present 3D visualizations for examiner interpretations. Several elements of a case, including the room, involved persons, weapons, etc., may be incorporated into a single animation to convey a comprehensive interpretation of the crime scene^43^. Ultimately, these animations may allow for a rapid overview of a complicated case. Finally, the post-mortem surface documentation can be used to automate autopsy procedures. Ebert et al. presented a robotic system which uses 3D data, generated by CT and photogrammetry, to guide a biopsy needle for minimally invasive autopsies^44^. The system is capable of needle placement with an accuracy of 3.2 mm. Although this technology was intended for use in the forensic setting, its implications may have an impact on clinical practice in the future.

## 5. Conclusions

There has been advances in several aspects of 3D data acquisition and visualization, largely due to improved performance and decreased costs of computers. Innovations in scanning and display methods have led to novel ways for the medical community to view and interact with 3D data, yet a majority of the technology has seen limited use in pathology.

Substantial progress has been made in the realm of macroscopic scanning techniques. However, several barriers in macroscopic specimen scanning need to be addressed before adoption for mainstream use. Since these scanning methods cannot be reimbursed, the costs of equipment need to decrease. Commercial scanners can range between a couple hundred to thousands of US dollars, with resolutions ranging between 0.06 mm and 1.5 mm^45^. The resolution of a majority of these scanners is not of diagnostic quality. Industrial scanners can reach high resolutions with precision; however, prices are in the tens of thousands of US dollars. Also, use cases must be further investigated to determine the value added by using 3D scanners. Validation studies are required to assess 3D scanning accuracy in object measurement and in addition to texture characteristics such as color.

Photogrammetry seems to be the best method to scan surgical specimens because it can obtain geometrical data of objects with relative accuracy and can generate models with photo-realistic quality. There is also the convenience of smartphones to capture this type of data, which are portable, relatively inexpensive, and ubiquitous. Mobile processors and digital camera sensors have seen consistent progress year after year, and thus smartphone capabilities in 3D scanning will continue to improve. Its restriction to surface scanning and high computing requirements are limitations to photogrammetry.

Interoperability also remains an issue for surface and volume data, as there are dozens of 3D model file formats (e.g., ASCII, OBJ, STL, PLY, 3DS, FBX)^1^. Both hardware manufacturers and software developers have failed to resolve this formatting issue. Each file format has their unique method of storing data, and not all file formats are capable of the same types of geometric or surface appearance characteristics, so conversion of file types leads to error or loss data^1^. This challenge is not an issue with volumes obtained from routine CT and MRI data sets because of DICOM standardization.

VR and AR are exciting developing technologies which have evolved dramatically in recent years. Its growth in popularity and applications in the military, sales, and medical fields show VR and AR are more than just a novelty. There is great potential for VR use in anatomic pathology, especially in macroscopic education and simulation. Fast graphics processors, high-resolution displays, and motion controllers now make it possible to develop a gross dissection simulator for resident training. This would be an immersive virtual environment where hand gestures or motion controllers are used to emulate specimen orientation, inking, sectioning, and visual inspection. A simulator would allow residents to repeatedly practice on challenging or rare specimens outside of the laboratory. Simulators have been used for surgery training with positive results^46^.

On the other hand, the greatest strength of AR is its ability to present digital content in the context of our surrounding environment. This empowers the user to receive computer assistance and retrieve on-demand information, while simultaneously performing tasks. Gross dissection may be enhanced by superimposing specimen-handling instructions onto surgical specimens. It can be used to assist fine-needle aspiration procedures overlaying radiologic images onto the patient. The possibilities of AR are endless, and its applications in anatomic pathology have barely been investigated.

Radiology has clear experience with 3D data, mostly due to their gradual transition from analog film to digital images at the turn of the century^47^. This is a great advantage radiology has had over pathology, in that volumetric data are routinely captured in the clinical setting. There is also the added benefit of DICOM standardization and scanning anatomic structures in-situ. The 3D data can be clinically applied in a myriad of ways before surgery with minimal harm to the patient. In contrast, anatomic pathology practice mostly remains analog, with few exceptions. Digital images have been adopted for macroscopic photography, telepathology, and consultation cases, but despite the supporting data on whole-slide imaging, routine use has not been widely accepted^48,49^. This is especially true in the United State, mostly due to the combination of Food and Drug Administration regulations, lack of whole-slide imaging system standardization, and pathologists’ views on digital sign out. Also, the creation of digital slides requires a glass slide to be produced, so the additional steps, equipment, and volume of slides required for routine 3D reconstructions raises concerns about turnaround time and return on investment.

A majority of the aforementioned examples of 3D data applications in anatomic pathology have limited supporting literature, low adoption rates, and varying success when implemented. These technologies deserve a greater attention because there is still much work to be done to investigate its utility and value. There is great potential for 3D technologies with innovations and novel applications yet to be discovered.

- **In anatomic pathology there is little research and development of this technology and is limited to educational and research applications
**
- **Additional innovations and refinements of current technologies are required to facilitate routine practical use of 3D data in the anatomic pathology laboratory
**
- **The utility of these emerging technologies remains unknown and will require investigation into the value it can bring to both clinical and nonclinical settings**
